# Supplementary material for: Transcription Factor SrsR (YgfI) Is a Novel Regulator for the Stress-Response Genes in Stationary Phase in Escherichia coli K-12
Source: Int J Mol Sci. 2022 May 27;23(11):6055. doi: 10.3390/ijms23116055 (PMC9181523; doi:10.3390/ijms23116055)
Supplement: Supplementary file 1 [file ijms-23-06055-s001.zip › ijms-1705889-supplementary.pdf]

Table S1. Primers used in this study.

(A) For gel shift assay

name	sequence
dinJ-F	CTGGTCATGCTTTAATGTTGCAGTG
dinJ-R	GGCCTTATGAACATCAATGCCAGC
appY-ompT-F	GTCACAGATGGAATAAACAAAACAATTGACTGA
appY-ompT-R	GCCCCGGGGAATAACTAGCC
dgcT-F	CCACCTGACCATTTTCATTGGTTAACTC
dgcT-R	TGTTCCACGATCAACCTATCTGATGG
ycgF-F	CTGGTTTGTTACTCCTGTCACGC
ycgF-R	TTTAGCTTTAGCAAACAGATCTCCCTGA
ycjM-F	CGGTTTGCGGGTCAACGG
ycjM-R	CCGATCAATTTCACAGCAATGATTTAAAAC
paaZ-A-F	GTACCGGATAAGAAACTGGCTAACTGC
paaZ-A-R	CACGAATCACATTGTTTTATGAAAGTTACAC
cdgI-F	CCATGGGGTTATTCTTTAAATATTTTTTATCGTTAAC
cdgI-R	GACTTTGTCTGGATCAGTGTACTGC
yfdO-P-F	ATCATCCTGCGTTCCCCACT
yfdO-P-R	AACCGCAATGCGTCGAATGT
fucA-P-F	CGTTCCATTAGCTACCTCTCTCTGA
fucA-P-R	AGAAGGTGACTTTATGTGACTACCATCAC
yjcF-F	TTTGCAGGTTGCAGACTACAAAACG
yjcF-R	TCAGGTTGACACGAGACAGATCA
lacUV5-F	CAGCTGGCACGACAGGTTTC
lacUV5-R	AGCTGTTTCCTGTGTGAAATTG

(B) For DNaseI footprinting analysis

name	sequence
yfdO/yfdP-F	GAACGCAGTACTGATGTG
yfdO/yfdP-R	GGCTCTGGAAAAAAATCAT

(C) For RT-qPCR analysis

name	sequence
srsR-F	TGCGAATATGCGACCTTGTG
srsR-R	ATTTCAGCGTTGCCGGATTC
yafQ-R	CCGCGTGAGTTCCAGTTCTC
yafQ-F	CGCTGCAAGGTTCATGGAAAG
yafL-F	GCAATGCCGAAAGCAAAAGG
yafL-R	AGCAGGAAACCATTCTGGATGC
paaZ-F	TTATTGTGCCGCAGGCATTG
paaZ-R	TTACCAGTGCGCCCATTTTC
paaA-F	GGTCGCGGAATTTGTAATCAGG
paaA-R	CGGGCATGTTGTTTTTGTGC
paaK-F	TTAATCCTCATGACGGCACACC
paaK-R	TGACCGGCAATGCTTCTTTG
cdgI-F	GGCGCTACTTTTTCAACAGC
cdgI-R	TTATCACCCACACGATGTCC
yfdO-F	GAAGCTGCGGGAAAAATG
yfdO-R	CGCGTTTTTCTGTGTACGTG
yfdK-F	TGGTAAAGCGGCTATTGGTC
yfdK-R	AATATCTGGCGCACTGGAAG
yfdP-F	AACGCAGTACTGATGTGCAG
yfdP-R	ACTGCGCCGATTTTGGTTTC
yfdQ-F	TCGAAGCGAAGACCAAAGAC
yfdQ-R	TCGCCAGTGATAATGCTGAG
fucA-F	TTGCACGGCAGTTTCCATTC

fucA-R	TTTTTGAGAGCCAGCGCAAC
fucO-F	TGATAAGGATGCCGGAGAAG
fucO-R	CAACCCTAACCCAACATTCG
fucP-F	TTATTCCATTCGCGCTGCTG
fucP-R	AAAAGGCCGATTGGATCAGG
fukR-F	ACGTCAATCCCTCGCTGATTTC
fucR-R	ATCAGCGTTGATCGCATTGG
rrsA-F	TGCATCTGATACTGGCAAGC
rrsA-R	TACGCATTTCACCGCTACAC

(D) For Northern blot analysis

name	sequence
yfdK-F	ATGAATTACATATATTCCGCGAC
yfdK-R	AGGAGTAGGCCATTCAATATC
